# The complete chloroplast genome sequence of *Angelica Tsinlingensis* (Apioideae)

**DOI:** 10.1080/23802359.2018.1436995

**Published:** 2018-04-23

**Authors:** Hui Zhang, Xiao-Fan Wang, Da Cao, Jun-Feng Niu, Zhe-Zhi Wang

**Affiliations:** a Key Laboratory of the Ministry of Education for Medicinal Resources and Natural Pharmaceutical Chemistry, Shaanxi Normal University, Xi’an, Shaanxi, P. R. China;; b National Engineering Laboratory for Resource Development of Endangered Chinese Crude Drugs in Northwest of China, Shaanxi Normal University, Xi’an, Shaanxi, P. R. China;; c College of Life Sciences, Shaanxi Normal University, Xi’an, Shaanxi, P. R. China

**Keywords:** *Angelica tsinlingensis*; complete chloroplast genome; Apioideae; Phylogeny

## Abstract

*Angelica tsinlingensis* is endemic in Shaanxi province (China), and is used as *Angelica sinensis* in folk. Owing to important medicinal value, wild *A. sinensis* has been over-exploited and become quite rare in China during over 2000 years. *Angelica* sensu lato (s.l.; *Apiaceae* subfamily Apioideae) is a taxonomically complex and controversial group, and *A. tsinlingensis* is clearly different from members of the *Angelica s.s.* clade with the morphological and molecular results. The complete chloroplast DNA sequence of *A. tsinlingensis* (GenBank accession number: MF924726) was determined in our study. The size of chloroplast genome of *A. tsinlingensis* is 147,104 bp, including a large single-copy (LSC) region of 93376 bp and a small single-copy (SSC) region of 17,574 bp separated by a pair of identical inverted repeat regions (IRs) of 18,077 bp each. A total of 125 genes were successfully annotated containing 83 protein-coding genes, 34 tRNA genes and 8 rRNA genes. GC content of IRs region is the highest (44.5%). The result of Phylogenomic analysis supports the difference of *A. tsinlingensis* from *Angelica s.s.* clade.

*Angelica tsinlingensis* is endemic in Shaanxi province (China), and is used as *Angelica sinensis,* a traditional Chinese herb used in female health related diseases, in folk (Li et al. 1993; Mazaro-Costa et al. 2010). In addition to its medicinal uses, *A. sinensis* is also used as a health food, a cosmetic and a dietary supplement in Asia, Europe and America (Filipiak-Szok et al. 2014). Owing to important medicinal value, wild *A. sinensis* has been over-exploited and become quite rare in China during over 2000 years (Zhang et al. 2012). *Angelica* sensu lato (s.l.; *Apiaceae* subfamily Apioideae) is a taxonomically complex and controversial group, exhibiting much morphological diversity and problematic generic limits. *A. tsinlingensis* is clearly different from members of the *Angelica s.s.* clade with its thin-winged dorsal ribs and triple vittae in each furrow and this discordance corresponds with the molecular results in placing *A. tsinlingensis* outside of the *Angelica s.s.* clade (Liao et al. 2013). In present study, the completed chloroplast genome sequence of *A. tsinlingensis* is reported contributing to conservation of this species and providing significant information for the complex taxon of Apioideae.

Fresh leaves of *A. tsinlingensis* were collected from greenhouse of National Engineering laboratory for Resource Developing of Endangered Chinese Crude Drugs in Northwest of China (108°53′30″E, 34°9′14″N; NSII accession number C072801, http://www.nsii.org.cn/2017/specimen.php?id =17426473) in Xi’an city. Those leaves were stored in the refrigerator at −80 °C. Total genome DNA of *A. tsinlingensis* was sequenced by Illumina Hiseq 2500 Sequencing System (Illumina, Hayward, CA) to construct the shotgun library. The low quality sequences were filtered out Using CLC Genomics Workbench v8.0 (CLC Bio, Aarhus, Denmark) and then reconstructed the chloroplast genome by using MITObim v1.8 (University of Oslo, Oslo, Norway; Kaiseraugst, Switzerland) (Hahn et al. 2013), with *Angelica acutiloba* (GenBank: NC_029391.1) as the reference. The complete chloroplast genome of *A. tsinlingensis* was annotated in Geneious R9 (v9.0.2) (Wang et al. 2017) and then submitted to GenBank (accession no. MF924726).

The size of chloroplast genome of *A. tsinlingensis* is 147,104 bp, including a large single-copy (LSC) region of 93376 bp and a small single-copy (SSC) region of 17,574 bp separated by a pair identical inverted repeat regions (IRs) of 18,077 bp each. A total of 125 genes were successfully annotated containing 83 protein-coding genes, 34 tRNA genes and 8 rRNA genes. GC content of the whole genome, IRs, LSC and SSC regions are 37%, 44.5%, 35.4% and 30.3%, respectively. GC content of IRs region is the highest. 18 genes contain one intron, while 2 genes have two introns.

The complete chloroplast genome sequence of *A. tsinlingensis* and other species from Apioideae were used to construct phylogenetic tree (Figure 1). A neighbour-joining (NJ) tree was performed with Mega 6.0 using 1000 bootstrap replicates (Tamura et al. 2013). The result shows the difference of *A. tsinlingensis* from *Angelica s.s.* clade, which is consistent with morphological and molecular results (Liao et al. 2013).

**Figure 1.**
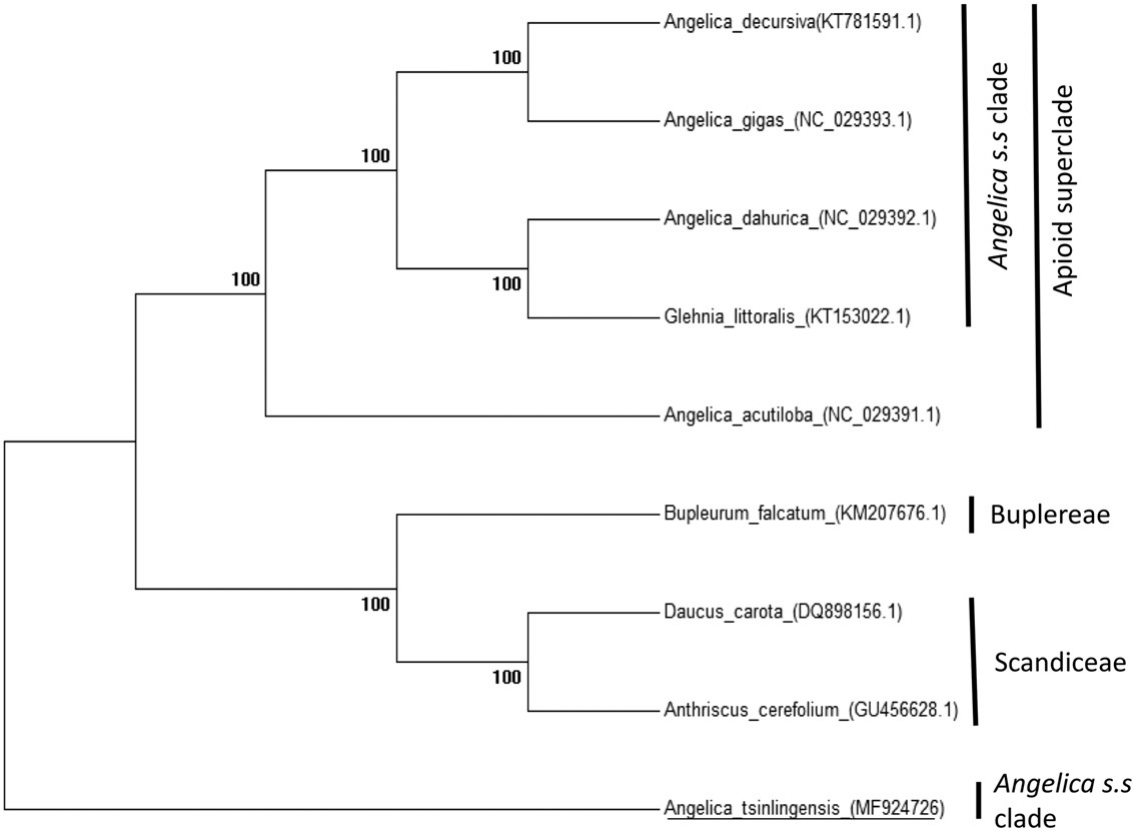
Phylogeny of nine species within the Apioideae based on the neighbour-joining (NJ) analysis of the complete chloroplast genome sequence. The gene’s accession number is list in figure.
